# Challenges and opportunities for control and elimination of soil-transmitted helminth infection beyond 2020

**DOI:** 10.1371/journal.pntd.0007201

**Published:** 2019-04-11

**Authors:** Matthew C. Freeman, Oladele Akogun, Vicente Belizario, Simon J. Brooker, Theresa W. Gyorkos, Rubina Imtiaz, Alejandro Krolewiecki, Seung Lee, Sultani H. Matendechero, Rachel L. Pullan, Jürg Utzinger

**Affiliations:** 1 Department of Environmental Health, Emory University, Atlanta, Georgia, United States of America; 2 Modibbo Adama University of Technology, Yola, Nigeria; 3 College of Public Health, University of the Philippines Manila, Manila, the Philippines; 4 Global Health, Bill & Melinda Gates Foundation, Seattle, Washington, United States of America; 5 Department of Epidemiology, Biostatistics and Occupational Health, McGill University, Montreal, Quebec, Canada; 6 Children Without Worms, The Task Force for Global Health, Decatur, Georgia, United States of America; 7 Instituto de Investigaciones en Enfermedades Tropicales, Universidad Nacional de Salta, Oran, Argentina; 8 Save the Children, Washington, DC, United States of America; 9 Ministry of Health, Nairobi, Kenya; 10 London School of Hygiene & Tropical Medicine, London, United Kingdom; 11 Swiss Tropical and Public Health Institute, Basel, Switzerland; 12 University of Basel, Basel, Switzerland; Imperial College London, UNITED KINGDOM

## Introduction

More than half of the world’s population lives in places endemic for soil-transmitted helminths (STHs), and an estimated 1.45 billion people are infected [1,2]. In 2017, the global burden of STH infection (*Ascaris lumbricoides*, hookworm, and *Trichuris trichiura*) was estimated at 1.9 million disability-adjusted life years (DALYs) [3]. Moderate and heavy infection intensity and chronic STH infection are associated with anemia, malnutrition, educational loss, and cognitive deficits, but recent systematic reviews and meta-analyses produced conflicting results on the impact of preventive chemotherapy (PC) [4–6].

The Soil-Transmitted Helminthiasis Advisory Committee (hereafter called “the Committee”) is a group of independent experts with a broad range of expertise. It is convened annually by Children Without Worms (CWW), an organization whose purpose is to utilize available evidence to identify best practices and opportunities for the prevention and control of STH infection [7]. On November 1 and 2, 2017, the Committee met in Baltimore, Maryland, United States of America, in order to discuss the critical need to develop a data-driven guide to the STH endgame on late-stage program functioning, processes, and surveillance. The focus was on research and field experiences from countries approaching the “elimination of STH infection as a public health problem” after consecutive years of PC and countries that are now considering scaling down their PC frequency but may be concerned about infection rebound. Emphasis was placed on interim recommendations for monitoring and decision-making for national program managers desiring to achieve the World Health Organization (WHO) goal of eliminating STH infection as a public health problem by 2020, particularly related to STH infections in risk groups other than school-age children (SAC), namely preschool-age children (PSAC) and women of reproductive age (WRA) [8]. The following is the Committee’s recommendations stemming from the Baltimore meeting in November 2017. It complements and updates the publication derived by the Committee’s meeting a year earlier in Basel, Switzerland [7], and was instrumental in shaping the agenda for the October 2018 meeting, convened jointly by CWW and WHO, with recommendations to be reported elsewhere.

## The 2020 roadmap and beyond

In its roadmap for implementation for 2020, WHO set a goal to achieve at least 75% coverage of PC—either annual or biannual—of SAC and PSAC [9–11]. As we approach 2020, it is imperative that we not only accelerate what has worked for the control of STH infection–related morbidity but that we look beyond 2020 and better understand what more is required to eliminate STH infection as a public health problem.

**Fig 1:**
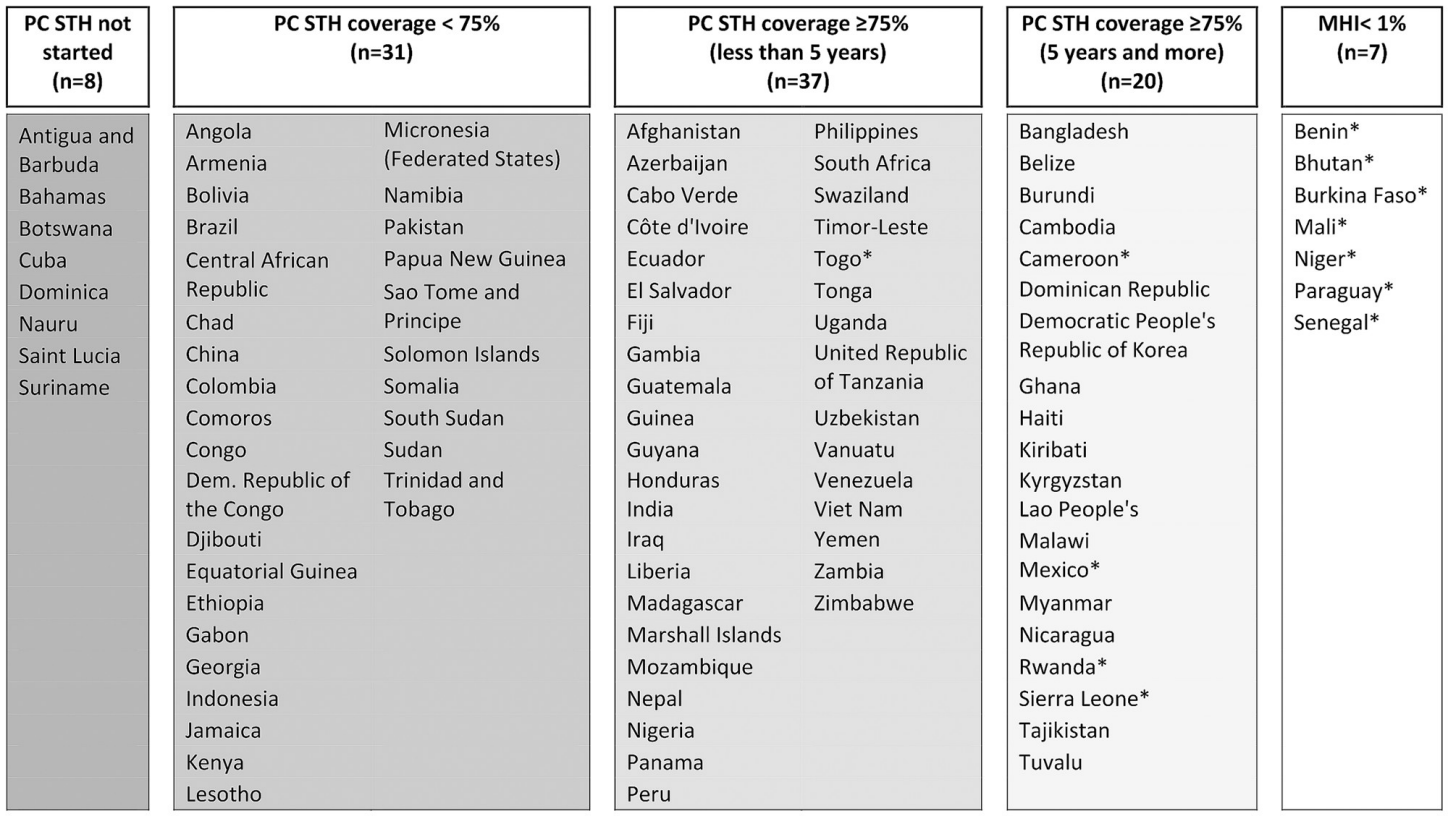
Progress for PC coverage (by country) in SAC and where STH infection is no longer considered a public health problem (MHI <1%). Source: 2016 PC data from WHO. * Country with detailed epidemiologic information available. MHI, moderate or heavy infection; PC, preventive chemotherapy; SAC, school-age children; STH, soil-transmitted helminth.

**Fig 2:**
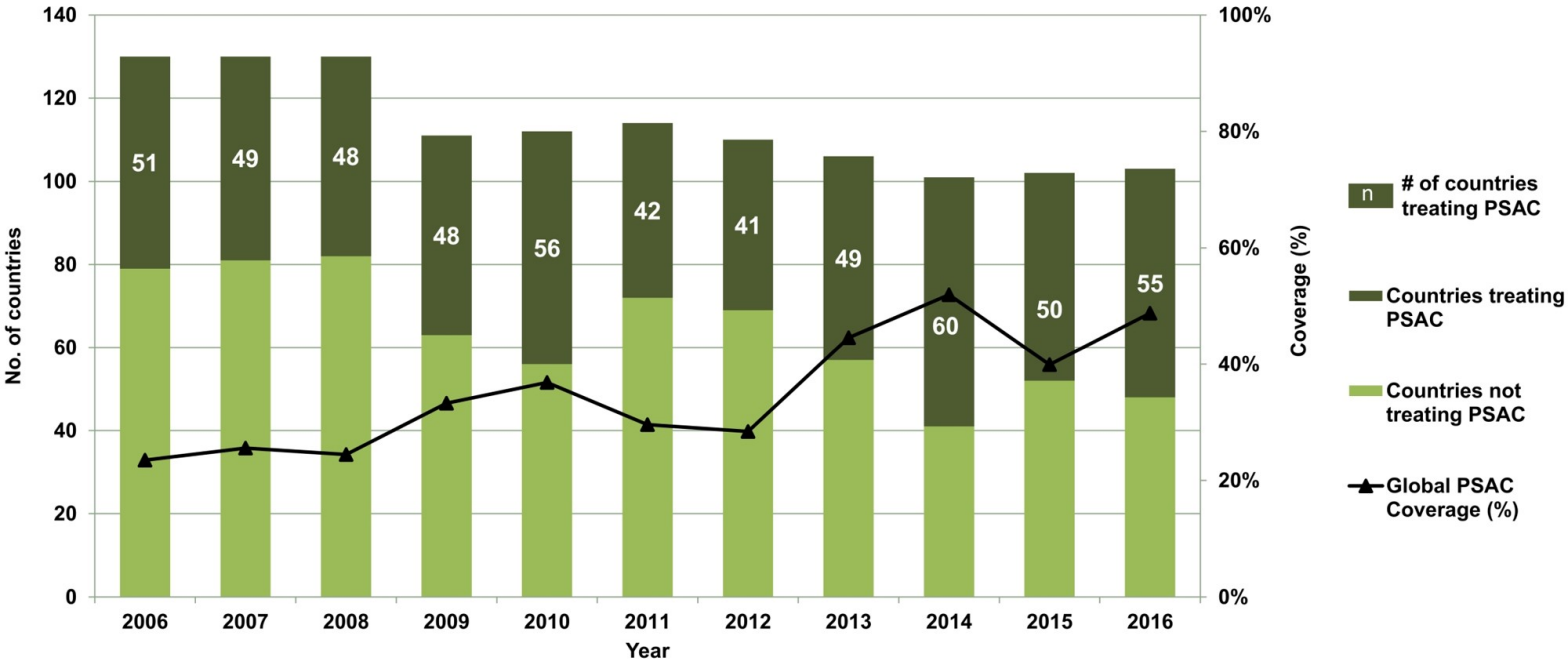
Global PSAC treatment and coverage, 2006–2016. Source: WHO PC Databank, PSAC PC coverage data from 2006–2016; http://www.who.int/neglected_diseases/preventive_chemotherapy/lf/en/. * Coverage is calculated by dividing the number of children requiring PC and treated by the total number of children in need of PC. PC, preventive chemotherapy; PSAC, preschool children; WHO, World Health Organization.

Fig 1 summarizes progress made in terms of both coverage and impact using 2016 country data. Despite considerable gains in SAC coverage, less than half of the at-risk countries are treating PSAC, a number that has not changed much in recent years, yielding a total coverage level of approximately 50% but with considerable variability of coverage from year to year and across countries (Fig 2). If the current trend in PC coverage persists, PSAC and the combined group of children aged 1–14 years will not reach the goal by 2020. Accelerating PSAC PC coverage might avoid that failure, and hence, needs a clear, strong global policy now. While the initial focus on SAC coverage has enabled the mobilization of resources, other priorities have emerged, both in places and populations in which the target was not achieved and within areas that are now shifting priority from scaling up PC to eliminating STH infection as a public health problem—defined by the WHO as when less than 1% of the at-risk population has moderate or heavy infection (MHI) [8]—and potentially interrupting the transmission of STH infection [12]. WHO recommends stopping PC once less than 1% of the at-risk population has MHI infections. Surveillance will, however, need to continue in order to pick up potential recurrence of infection and to plan further intervention, if warranted. Hence, as we turn our sights towards and beyond 2020, the Committee recognized it as timely to review and assess the successes and challenges of progress made to date. Indeed, it is hoped that the Baltimore 2017 meeting deliberations will inform future control strategies and targets.

There have been many important developments by the time the Committee convened in November 2017, but six are particularly noteworthy:

- updated PC guidelines published by WHO for all at-risk populations [13];
- the Bellagio Declaration focusing on girls and WRA [14];
- widening of WHO’s engagement with experts around the globe [15];
- success of the Global Program to Eliminate Lymphatic Filariasis (GPELF) [16];
- increasing importance for deworming programs to measure impact [17]; and
- the launch by WHO-AFRO of the Expanded Special Project on the Elimination of Neglected Tropical Diseases (ESPEN) portal (http://espen.afro.who.int), which provides—for the first—subnational data on disease endemicity and PC coverage for each of the five PC-NTDs, including STH infection.

In this Policy Platform, we lay out critical challenges in seven key areas that need attention, discuss progress until November 2017, and put forward recommendations for immediate action. Our recommendations arose at a critical juncture for STH control efforts, as current global policies, goals, and related strategies and resources are revitalized through 2020, a year which is upon us.

## Challenges and recommendations

### Challenge #1: Incomplete and inconsistent monitoring of program impact

#### Recommendation: Define standard impact goals and targets post-2020

To date, programs mainly focused on reporting on PC coverage, as guided by a simplified coverage target specified by the Roadmap; yet there are few standardized data on program impact. It is noteworthy that country programs are increasingly interested in quantifying the impact of deworming on health outcomes [17–19]. Current approaches to measuring impact vary across countries and across deworming program implementers, limiting comparability and the possibility to appreciate changes over time and across countries and regions. Moreover, impact data are not readily available, with most of the evaluations conducted by research groups. For the Africa region, there has been recent progress in data sharing and transparency for neglected tropical diseases (NTDs), through the work of ESPEN and their data portal [20]. Currently, there is availability of data on endemicity and coverage at the subnational level for the five PC-NTDs, including STH infection, for 47 of 49 countries in the AFRO region. This successful approach to reporting sub-national data should be expanded to the other WHO regions where STHs are endemic.

The ESPEN portal is starting to include impact assessment data for lymphatic filariasis and onchocerciasis and there is an opportunity to include comparable data for STH infection. However, this will require a standardized and comprehensive monitoring and evaluation (M&E) framework that is tied to clearly defined, quantitative goals. For example, globally, a reported 69.5% of SAC and 50.8% of PSAC requiring PC reportedly received PC in 2016 [21]. It is conceivable that the observed scale-up of PC targeting STH over the past decade was a major contributor to the decline in the global burden of STH infection [22–25]; yet the precise extent to which this coverage has reduced prevalence, intensity, and burden of STH infection is not known. Country programs, in particular, require a comprehensive, standardized, yet flexible approach to measure progress toward morbidity-related goals. Such an approach would capture essential programmatic elements, and be used by each country to map their needs, commitment, and resources. This will generate realistic timelines and planning processes as well as alerting WHO, partners, and donors to better assess the resource and technical capacity needs of each program. Subnational data with standardized indicators for anthelmintic drug availability and coverage of the target populations are critical to track progress at the subnational level where PC program implementation may not be uniform. Data can also be disaggregated (e.g., by district, sex, and age categories, and any other useful determinant) to better understand the equity of program access and delivery for the three target risk groups (i.e., PSAC, SAC, and WRA). PC needs for refugees and migrants are also increasingly being recognized, and hence, WHO is considering to add these populations to the at-risk groups, while some countries (especially those in AFRO and EMRO regions) are actively engaged in estimating access, burden, and resource issues for migrants.

### Challenge #2: Reaching at-risk groups other than SAC

#### Recommendation: Identify new PC strategies, platforms, and reporting mechanisms

Deworming of SAC has been shown to reduce disease burden, especially reducing high-burden infections in a cost-effective manner, but the empirical evidence from both multiyear deworming programs and modeling studies suggest that targeting SAC alone for PC is insufficient for sustained control and elimination of STH infection [17,26,27]. In particular, this is the case for *T*. *trichiura* due to poor drug efficacy [28,29] and for hookworm due to the age distribution of infection. Indeed, in many settings, adults are at particularly high risk of hookworm infection and thus contribute substantially to transmission [30]. These issues are in contrast to countries where school-based deworming has been coupled with community-based programs in both experimental studies and as part of lymphatic filariasis control programs that target entire communities [31]. Using an exclusive school-based intervention platform potentially excludes 12.8% SAC who are out of school, with some countries like South Sudan having 66% of their SAC out of school [32]. With that and the availability of a pediatric mebendazole preparation as a donation for PSAC, out-of-school children and PSAC can be immediately prioritized to help achieve the overall children’s coverage goal. Recent progress has been made on a more comprehensive preventive approach to include all risk groups, as demonstrated by the Kenya “Breaking Transmission Strategy” in addition to community-wide PC coverage through the lymphatic filariasis elimination program through USAID. Successful PC interventions targeting SAC by most countries are generating requests for specific guidance on the next operational phase: how to efficiently implement sentinel surveillance and which indicators to measure in order to predict, detect, and treat widely dispersed and persistent pockets of transmission. There is a need to consider how to scale up approaches to reach PSAC, given the current drug availability and added cost of this approach. Consistent with these needs is a recognized gap in our current knowledge of disease transmission at low prevalence and persistent environmental factors that facilitate transmission. Thus, there is a renewed need to clearly identify research gaps and questions that would facilitate implementation in these settings.

A recent report has provided guidance and recommendations for WRA [33]. Building on this report, WHO needs to develop implementation guidelines linked to clearly defined targets, both for WRA and PSAC as critical populations at risk of high STH burden [7,14]. Operational research is also needed to define platforms, partners, and recording and reporting tools to monitor progress of control programs targeting PSAC and WRA. In addition, there remains the challenge of providing additional anthelmintic drugs necessary to treat WRA (not targeted by the current donations). There is optimism that there will be the possible donation of chewable Vermox from Johnson & Johnson (J&J) for PSAC, but the quantities donated may not be enough to cover the total numbers at risk. Consideration of new drug options for WRA needs immediate discussion at national, regional, and global fora. Data from the GPELF and other community-based PC programs may provide insight into efficacy and safety issues through birth cohort studies.

### Challenge #3: The risk of anthelmintic drug resistance

#### Recommendation: Develop standardized indicators to detect emerging resistance

Experience from the veterinary sector demonstrated that anthelmintic drug resistance developed after years of large-scale monotherapy [34]. We suspect that if we wait until resistance is clinically detected in humans, it will be too late to respond [35]. While progress is being made, there are currently no routine, field-applicable diagnostics that can effectively identify and monitor signs of emerging resistance, so research on developing such tests urgently needs financial support. Indeed, we need to better identify resistant genes and to track refugia (i.e., that proportion of the worm population that remains susceptible to anthelmintic drugs). Human populations with a long history of single-drug deworming that have low worm burdens but have not reached transmission break points are likely to be at the greatest risk for the development of drug-resistant parasites. For diagnostic approaches such as quantitative polymerase chain reaction (qPCR) to be used in an STH-programmatic setting, we need to take molecular diagnostics to a level at which we can use its full potential by standardizing analysis and reporting and including appropriate quality control measures [36]. A standardized approach to the monitoring of potential emerging resistance needs to be established, especially in those countries that have mature PC programs (e.g., Mexico and Togo, among others), to track both drug efficacy and mutations known to be associated with resistance [37].

### Challenge #4: Poor diagnostics to assess program needs by implementation stage

#### Recommendation: Employ validated program stage-specific diagnostic techniques

There is a need for new diagnostics that are appropriate for informing key decision points for national STH control programs [38,39]. The Kato–Katz technique, while relatively inexpensive, widely used, and sensitive in detecting MHI, will have lower positive predictive values in low-prevalence (and low-intensity) settings [40,41]. In addition, some preliminary analysis from microscopy and PCR has suggested that hookworm infection may be misidentified. New diagnostic tests have been validated [7], yet there is a need for novel, highly sensitive tests that can be employed in settings that move from STH control to elimination. However, their specific role and use in the context of national program implementation needs to be assessed [41]. Furthermore, capacity strengthening for national programs will be needed to fully take advantage of any new diagnostics. Field and laboratory protocols need to be standardized, reference laboratories established, and training developed for different contexts and languages. Gaps in the current diagnostic landscape have been identified, and formative research is underway. The next steps are to finalize and disseminate results and to identify additional resources for gap areas (e.g., sustaining animal models, conducting field studies to calculate test performance in various settings, etc.). Guidelines will then need to be developed to inform country programs on how to incorporate these tests for improved assessment of program impact and further planning.

### Challenge #5: Limited efficacy of current drugs and gaps in drug availability

#### Recommendation: Promote research into combination therapies and fast track pre-qualification processes

The existing anthelmintic drugs have variable efficacies against different STH species, with particularly low efficacies against *T*. *trichiura* when using single-dose treatments [29]. New efforts must be undertaken to identify and provide guidelines for use cases for combination therapies in general and specifically in which *T*. *trichiura* is the predominant species [42]. In 2018, the WHO Essential Medicines Committee approved the inclusion of ivermectin for both STH and *Strongyloides stercoralis* to the WHO Model List of Essential Medicines [43]. However, there are implications and potential challenges in adding ivermectin to albendazole and mebendazole in STH deworming programs. Merck provides ivermectin dedicated for the control of lymphatic filariasis and onchocerciasis, and new manufacturers will need to become prequalified to meet the growing demand from STH control programs. Additionally, bioequivalence studies and other considerations will be needed to make the drug available at low cost. This will take time and requires innovative financing mechanisms.

### Challenge #6: Limited coordination with the water, sanitation, and hygiene (WASH) sector

#### Recommendation: Identify WASH indicator(s) to be included in routine STH M&E

As articulated in an editorial put forth in *The Lancet* [44], the 2020 WHO roadmap identified the critical role of WASH in the control of STH infection but did not set actionable targets or strategies [11]. The STH community has largely avoided establishing an approach for addressing WASH or establishing a structure for engagement with the WASH sector. However, the recent development of the WASH–NTD joint strategy provides an entry point and guidance for improved communication, coordination, and collaboration [45]. Ample observational evidence [46,47], some recent randomized trials, biological plausibility, and history suggest that improved WASH is critical in the control and elimination of STH infection. Alignment with the WASH sector, specifically including WASH indicators as part of STH M&E [48] (and vice versa) by using data to advocate for WASH activities in STH-endemic areas, employing program monitoring, and conducting operational research and advocacy to ensure normative inclusion of STH-related WASH behaviors (e.g., shoe-wearing [49]), would be valuable contributions in STH control programming. Better quantification of the specific mechanisms and use of consistent indicators across programs would provide support to the WASH sector on gaps in typical WASH programming (e.g., type of water sources or food hygiene).

### Challenge #7: New targets needed for post-2020

#### Recommendation: Develop clearly defined, quantitative program targets for all at-risk groups and move beyond PC coverage estimates

One of the most important next steps as we move toward 2020 and beyond is the need to establish clearly defined, quantitative program goals and targets post-2020. The current targets of achieving at least 75% coverage for deworming for SAC (and PSAC) is in reach, but we know that even meeting this target is insufficient to achieve elimination of STH as a public health problem. We must also critically evaluate whether the threshold of 1% prevalence of MHI is useful for M&E of STH morbidity control and moving toward elimination. There is a need to look beyond simple PC coverage measures and include impact targets for PSAC and WRA, benchmarks for WASH that will encourage investment in WASH in STH-endemic areas, and estimates of PC uptake along the distribution chain that would give accurate estimates for not only availability of the drug, but population compliance. Addressing Challenges 1–6, articulated above, will support this critical effort.

## Outlook

There has been substantial progress in increasing coverage of PC for the control of STH infection, particularly among SAC and, to a lesser extent, among PSAC. As we approach 2020, work remains to accelerate action to achieve these targets in many places. At the same time, we need to think critically about what is needed beyond the 2020 roadmap and increase efforts in the seven areas discussed in this Policy Platform. This needs to be achieved through active collaboration and coordination by pertinent government ministries, researchers, donors, WHO, drug manufacturers, and multisectoral collaboration [50]. In doing so, it will help ensure progress toward eliminating STH infection—and other NTDs—as a public health problem, and it will yield more efficient allocation of resources and greater sustained impact, driven by targets and thresholds based on scientific evidence.
